# Low dietary sodium potentially mediates COVID-19 prevention associated with whole-food plant-based diets

**DOI:** 10.1017/S0007114522002252

**Published:** 2023-04-14

**Authors:** Ronald B. Brown

**Affiliations:** School of Public Health Sciences, University of Waterloo, Waterloo, ON N2L3G1, Canada

**Keywords:** COVID-19 prevention, Low sodium diet, Sodium toxicity, Whole-food plant-based diet, Sodium:potassium ratio, Nutritional epidemiology, Nutritional immunology

## Abstract

Compared with an omnivorous Western diet, plant-based diets containing mostly fruits, vegetables, grains, legumes, nuts and seeds, with restricted amounts of foods of animal origin, are associated with reduced risk and severity of COVID-19. Additionally, inflammatory immune responses and severe acute respiratory symptoms of COVID-19, including pulmonary oedema, shortness of breath, fever and nasopharyngeal infections, are associated with Na toxicity from excessive dietary Na. High dietary Na is also associated with increased risks of diseases and conditions that are co-morbid with COVID-19, including chronic kidney disease, hypertension, stroke, diabetes and obesity. This article presents evidence that low dietary Na potentially mediates the association of plant-based diets with COVID-19 prevention. Processed meats and poultry injected with sodium chloride contribute considerable amounts of dietary Na in the Western diet, and the avoidance or reduction of these and other processed foods in whole-food plant-based (WFPB) diets could help lower overall dietary Na intake. Moreover, high amounts of K in plant-based diets increase urinary Na excretion, and preagricultural diets high in plant-based foods were estimated to contain much lower ratios of dietary Na to K compared with modern diets. Further research should investigate low Na in WFPB diets for protection against COVID-19 and co-morbid conditions.

A recent systematic review found limited and inconsistent evidence that treating micronutrient deficiencies with high-dose micronutrient supplements, including vitamins A, C, D, and E, and Fe, Zn, and Se, is effective in preventing and hastening recovery from diseases like COVID-19^(1)^. The researchers suggested that maintaining a balanced diet is more important for prevention of COVID-19. Furthermore, obesity and type 2 diabetes prevalence related to consumption of a Western diet high in refined foods increases a population’s risk for COVID-19 severity and mortality^(2)^. Alternatively, plant-based diets that include fruits, vegetables, grains, legumes, nuts and seeds, while restricting foods from animal origins such as meat, dairy products and eggs, are proposed to prevent human disease, as well as reduce environmental damage and eliminate animal suffering associated with an omnivorous Western diet^(3)^. Properly balanced plant-based diets provide adequate sources of protein, Ca, Fe, vitamin D and vitamin B_12_^(4)^.

Consumption of a Western diet leads to chronic inflammation by stimulating the innate immune system and impairing adaptive immunity, which reduces defences against viral infections^(2)^. Neuroinflammatory mechanisms associated with peripheral inflammation in COVID-19 are also exacerbated by a poor diet, which can lead to neurodegenerative disease and dementia. Additionally, obese patients with chronic low-grade inflammation related to high adiposity are more susceptible to severe SARS-CoV-2 infection, as are malnourished patients, especially the elderly who lack immune protection provided by a balanced diet^(5)^. During the outbreak of the COVID-19 pandemic in Wuhan, China, 52·7 % of infected patients aged 65 years and older were malnourished and an additional 27·5 % were at risk for malnutrition^(6)^.

‘In both undernutrition and obesity, the ingestion of monotonous diets rich in ultra processed foods may lead to vitamin and mineral deficiencies, impairing the immune system and increasing susceptibility to SARS-CoV-2’^(5)^.

An analysis of COVID-19 patients’ nutritional status in an Iranian population found that low fruit and vegetable intake was significantly associated with disease severity – 63 % of patients consumed between zero to one serving of fruit a day and 77 % reported no daily intake of vegetables^(7)^. On the other hand, the Mediterranean diet and Dietary Approaches to Stop Hypertension (DASH) promote dietary patterns rich in unprocessed fruits and vegetables, and these dietary patterns are associated with anti-inflammatory outcomes^(5)^. A systematic review and meta-analysis of seventeen randomised controlled trials found that adherence to the Mediterranean diet decreased inflammatory markers of endothelial dysfunction and CVD risk, such as C-reactive protein, IL-6 and intracellular adhesion molecule-1^(8)^. The Mediterranean diet is also associated with a protective effect against risk of COVID-19^(9)^. Although research on the DASH diet and COVID-19 is lacking, the DASH diet is noted for reduced inflammation and lower cardiac injury and strain when combined with low Na consumption^(10)^.

Compared with plant-based diets, the Western diet contributes additional sources of Na from meat and poultry. Na is added to processed meats, and sodium chloride is injected into poultry during processing to improve texture and water-holding capacity^(11)^, which can increase the Na content of a four-ounce serving of chicken from 50–75 mg to 400 mg^(12)^. A Canadian study found that Na was an added ingredient in 72 % of poultry products^(13)^. Next to pizza consumed by young people between 6 and 19 years of age, and yeast breads consumed by people 51 years and older, chicken and chicken mixed dishes provided the most Na of all food categories among all age groups in an analysis of data from the US National Health and Nutrition Examination Survey (NHANES) 2003–2008^(14)^.

A recent systematic review of global nations found that ultraprocessed food was most commonly consumed in the USA and Great Britain, with the least consumption in Italy, which the researchers attributed to the Italian population’s greater adherence to a traditional Mediterranean diet^(15)^. In spite of this, Italy has experienced a high rate of COVID-19 infections^(16)^. However, Italy is also known to have high dietary salt intake^(17)^. Consumers may add salt to whole-food recipes prepared with few or no foods processed with Na, and people often employ salt as a condiment at meals, emphasising an important point that not all whole-food diets are low in Na. Additionally, an Action on Salt survey of plant-based and vegan foods served in UK restaurants and fast-food outlets found a high level of salt in most items^(18)^. Dietary Na intake in various cultures and nations needs to be considered in research investigating the association of COVID-19 prevention with whole-food plant-based diets (WFPB).

The present article proposes that COVID-19 prevention associated with a WFPB diet is potentially mediated by high intake of unprocessed fruits, vegetables, whole grains, legumes, nuts and seeds, with low intake of ultraprocessed foods, Na and foods of animal origin. A grounded theory method was used when writing this paper to search and select relevant research findings from the peer-reviewed literature on dietary Na, WFPB diets and COVID-19^(19)^. A comparative analysis of the selected findings was used to conceptualise themes and synthesise the present grounded theory narrative.

## Sodium toxicity and COVID-19

### Sodium toxicity and COVID-19 symptoms

Na toxicity is a condition within the body caused by poisonous effects of excessive Na intake, as in overdose death from acute salt poisoning^(20)^. Na toxicity can also occur from large amounts of Na ingested over long periods^(21)^. A daily Na dietary intake greater than 2300 mg is associated with increased risks of CVD and other chronic diseases^(22)^. Furthermore, Na toxicity has been associated with the nutritional epidemiology and nutritional immunology of COVID-19^(23)^. The effects of Na toxicity on mechanisms of COVID-19 symptoms are summarised in this section.

Acute respiratory distress syndrome is a fatal condition in COVID-19 that blocks the air sacs of the lungs with a gummy yellow fluid causing severe shortness of breath and reduced arterial oxygen saturation^(24)^. Of relevance, lung fluid in acute respiratory distress syndrome appears identical to lung fluid in pulmonary oedema^(25)^. Recent research confirms that pulmonary oedema is a pathological feature in severe cases of COVID-19^(26)^. Furthermore, infusions of excess sodium chloride into the lungs have been found to cause pulmonary oedema^(27)^ – potentially relating acute respiratory distress syndrome and pulmonary oedema in COVID-19 to effects of Na toxicity from excessive sodium chloride and retained fluid that may accumulate in the lungs.

Other symptoms of COVID-19 include nasal sinus congestion, headache and fever which are also associated with Na toxicity. According to the WHO, sinuses in acute sinusitis are blocked with fluid which can cause headaches^(28)^. Fluid blockage in sinuses may be due to hypervolemia and nasal mucosal oedema from Na toxicity. Migraine headache pain is also associated with COVID-19^(29)^, and Na permeability through the blood–cerebral spinal fluid barrier as well as through the blood−brain barrier are increased in migraine^(30)^. Fever is listed among adverse effects of pharmaceutical sodium chloride tablets^(31)^. Furthermore, fever occurs from the pyrogen action of sodium chloride injected into laboratory animals, which may affect control of hyperthermia due to an imbalance between Ca and Na ions in the anterior hypothalamus^(32)^.

Virion aggregates are normally shed through the upper nasal passages of the mucosal immune system, but aggregates of SARS-CoV-2 may accumulate in the upper nasal passages due to impaired mucociliary clearance from the ciliostasis effect of sodium chloride^(33)^. Prolonged mucociliary clearance was found in COVID-19 patients compared with other patients with non-nasal symptoms^(34)^. Moreover, viral sepsis occurs as the accumulation of viruses and other particles in the upper nasal passages enter the bloodstream^(35)^, and sepsis was increased in elderly patients with increased concentrations of plasma Na in hypernatremia^(36)^. A link between sepsis and excess Na could explain the occurrence of sepsis in COVID-19 patients potentially related to Na toxicity^(35)^.

### Sodium toxicity and COVID-19 immnue response

Na toxicity also affects immune response mechanisms in COVID-19, summarised as follows. Changed immune responses that promote inflammation and organ damage are associated with sodium chloride intake, which increase the release of inflammatory cytokines: TNF-*α*, IL-6 and macrophage inflammatory protein-2^(37)^. Of relevance, responses to RNA viruses like SARS-CoV-2 are similarly associated with increased secretion of cytokines, IL-6 and TNF-*α*, with fewer antiviral responses and more pro-inflammatory responses^(38)^.

Increased levels of sodium chloride stimulate T-cell proliferation and lower anti-inflammatory responses – pro-inflammatory M1 macrophages are increased and anti-inflammatory M2 macrophages are suppressed by increased levels of sodium chloride. Moreover, sodium chloride enhances IL-4 and IL-13 production and suppresses interferon-γ (IFN-γ) produced in memory T cells^(39)^. Furthermore, clearance of extracellular pathogens is assisted by IL-17-producing helper T cells (Th17), and increased levels of sodium chloride activate a kinase signalling pathway that induces development of Th17 cells^(40)^.

Of significance, human receptor angiotensin-converting enzyme 2, a binding site for SARS-CoV-2, is found in alveolar macrophage cell membranes in the respiratory tract and in other immune system cells^(41)^, implying that angiotensin-converting enzyme 2 provides a protective mechanism in the endocytosis and lysis of pathogens. But this protection could be reduced as angiotensin-converting enzyme 2 expression is lowered by high sodium chloride dietary intake, as demonstrated in laboratory animal experiments^(42)^.

## Plant-based diets and COVID-19

Plant-based diets that are high in unprocessed fruits, vegetables, grains, legumes, nuts and seeds and low in animal-based food products were associated with reduced severity of COVID-19 in a case–control study of frontline healthcare workers from six countries^(43)^. Compared with workers who reported following a plant-based diet, higher amounts of meat and poultry consumed by other workers was associated with several fold greater odds of moderate to severe COVID-19. Healthcare workers who did not follow a plant-based diet consumed 24 % more fish and seafood, 91·6 % more poultry, and 192·3 % more red and processed meats than other workers. Researchers of the study concluded that future studies are needed with more detailed data of macro- and micronutrient dietary intake associated with COVID-19 severity^(43)^, which includes dietary intake of Na and K.

A randomised controlled trial found that participants assigned to dietary patterns categorised as vegan (no animal-based foods), vegetarian (permits eggs and dairy products) and pescovegetarian (permits seafood) had lower Dietary Inflammatory Index (DII) scores compared with participants assigned to semivegetarian and omnivorous dietary patterns^(44)^. Importantly, although all groups followed diets high in Na, intake of dietary Na reported at the end of the 6-month trial was lowest in the vegan group that consumed no meat, poultry or seafood.

More recently, a healthy diet was assessed using a Plant-Based Diet Score, and the diet was associated with lower risk and severity of COVID-19 in participants from the COVID-19 Symptom Study^(45)^. The healthful Plant-Based Diet Index (hPDI) used in the study places emphasis on fruits, vegetables and whole grains. The association of a poor diet with COVID-19 risk in the study was stronger among people of lower socio-economic status (SES). Relatedly, a systematic review and meta-analysis of socio-economic determinants of dietary Na intake in high-income countries found that ‘people of low SES consume more Na than do people of high SES’^(46)^. This implies that the increased risk and severity of COVID-19 in people with lower SES is associated with a poor diet that may also be high in Na.

However, in addition to diet, many social determinants are also associated with risk of COVID-19, such as reduced access to healthcare services, crowded environments with greater exposure to the coronavirus, and stress and co-morbid conditions associated with poverty^(47)^. Additional social determinants include food insecurity, unavailability of healthy and affordable food, unemployment, discrimination, crime and violence, lack of quality education, and poor health literacy^(48)^. More research is needed to investigate how social determinants interact with high-Na dietary patterns associated with COVID-19 risk in people with lower SES.

### Sodium:potassium ratio

High intake of dietary K, which is abundant in fresh fruits and vegetables, was found to counter effects of high salt intake associated with hypertension and inflammation^(49)^. K induces natriuresis, or Na excretion by the kidneys, and a low Na:K urinary excretion ratio is more strongly associated with low blood pressure than Na or K levels alone^(50)^. Compared with modern diets, preagricultural diets that emphasised whole plant-based foods were estimated to have much lower dietary Na:K ratios, providing preagricultural humans with approximately 600 mg of Na and 7000 mg of K a day^(51)^. WFPB diets are not the only source of adequate K – health authorities encourage the general public to consume five to six servings of fruits and vegetables a day to meet K needs^(52)^. Nevertheless, considering modern global food consumption patterns that favour high salt intake and low K, implementing dietary guidelines for Na and K from the WHO (under 2000 mg Na and at least 3510 mg K for adults per d) ‘will be an enormous challenge for global public health’^(53)^.

Modern Na dietary intake is higher than K intake in China^(54)^, and metabolic syndrome in Chinese adults is associated with a higher dietary Na:K ratio^(55)^. Moreover, hypokalemia (serum K < 3·5 mEq/l) was highly prevalent in patients hospitalised with COVID-19 in Italy^(56)^, China^(57)^ and Spain^(58)^. Likewise, hyponatremia is also common in COVID-19 patients (serum Na < 135 mEq/l), but more research is needed to determine whether the type of hyponatremia associated with COVID-19 is hypervolemic hyponatremia caused by water retention related to excess Na^(23,59)^. Interactions between Na and K in WFPB diets should be investigated in the prevention of COVID-19.

## Plant-based diets, sodium and COVID-19 co-morbidities

Severe cases of COVID-19 are often co-morbid with chronic kidney disease^(60)^, hypertension^(61)^, and stroke^(62)^, and plant-based diets and dietary Na restriction have been found to be effective in the prevention of these diseases and conditions. Plant-based diets are recommended for prevention and management of chronic kidney disease^(63–68)^, and dietary Na restriction has also been found beneficial in chronic kidney disease^(69–72)^.

Hypertension is effectively managed by low Na diets^(73–75)^ as well as by plant-based diets^(76–78)^. Na restricted diets have also been associated with reduced risk of stroke^(79,80)^, as have plant-based diets^(81)^. However, an exception to decreased stroke risk occurs in association with plant-based diets of low quality^(82)^, possibly related to salt additives in processed foods like refined grain products and snack foods.

Other co-morbidities of severe COVID-19 include diabetes^(83)^ and obesity^(84)^, and plant-based diets are associated with reduced risk of diabetes^(85–87)^ and obesity^(88,89)^. High dietary Na is also associated with diabetes^(90,91)^ and obesity^(92,93)^. Collectively, the above-cited studies suggest an association between Na, plant-based diets, and diseases and conditions co-morbid with severe COVID-19.

### Conclusions

**Fig. 1.**
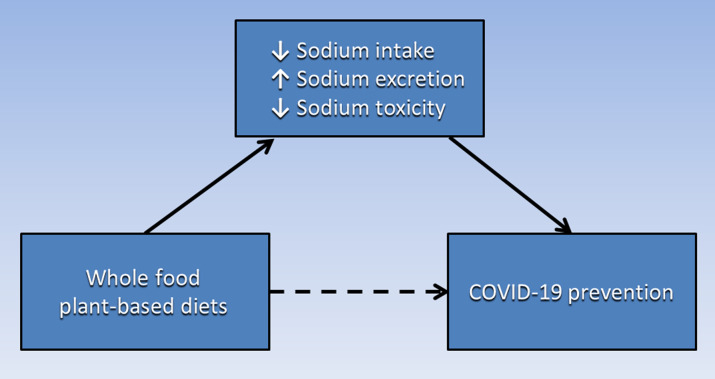
The association of WFPB diets with COVID-19 prevention (dashed arrow) is potentially mediated by low sodium intake, increased sodium excretion and reduced sodium toxicity. WFPB, whole-food plant-based.

Based on the evidence presented in this paper, Fig. 1 proposes a causative pathway in which the association of WFPB diets with COVID-19 prevention (dashed arrow) is potentially mediated by the combination of low Na intake, increased Na excretion and reduced Na toxicity. Future studies should examine the Na content of WFPB diets and the impact of reduced dietary Na on prevention of COVID-19 and co-morbid diseases. Additionally, the interaction of Na and K and the dietary Na:K ratio in WFPB diets should be further investigated for the prevention of these diseases. Finally, other components of WFPB diets may also contribute causative pathways to COVID-19 prevention, such as high fibre and low fat content, as well as vitamins, minerals and phytonutrients. These additional WFPB diet components should be investigated separately and in combination with Na and K.
